# Comprehensive Identification of Immunodominant Proteins of *Brucella abortus* and *Brucella melitensis* Using Antibodies in the Sera from Naturally Infected Hosts

**DOI:** 10.3390/ijms17050659

**Published:** 2016-04-30

**Authors:** Gamal Wareth, Murat Eravci, Christoph Weise, Uwe Roesler, Falk Melzer, Lisa D. Sprague, Heinrich Neubauer, Jayaseelan Murugaiyan

**Affiliations:** 1 Institute of Animal Hygiene and Environmental Health, Centre for Infectious Medicine, Freie Universität Berlin, Robert-von-Ostertag-Str. 7-13, Berlin 14163, Germany; Gamal.Wareth@fli.bund.de (G.W.); Uwe.Roesler@fu-berlin.de (U.R.); 2 Friedrich-Loeffler-Institut, Federal Research Institute for Animal Health, Institute of Bacterial Infections and Zoonoses, Naumburger Str. 96a, Jena 07743, Germany; Falk.Melzer@fli.bund.de (F.M.); Lisa.Sprague@fli.bund.de (L.D.S.); Heinrich.Neubauer@fli.bund.de (H.N.); 3 Faculty of Veterinary Medicine, Benha University, Moshtohor, Toukh 13736, Egypt; 4 Institute of Chemistry and Biochemistry, Freie Universität Berlin, Thielallee 63, Berlin 14195, Germany; eravci@zedat.fu-berlin.de (M.E.); chris.weise@biochemie.fu-berlin-de (C.W.)

**Keywords:** *Brucella*, host specificity, mass spectrometry, Liquid chromatography–mass spectrometry (LC-MS), two dimensional electrophoresis, 2D-PAGE, Matrix-assisted laser desorption/ionization-Tine of Flight-Mass Spectrometry MALDI-TOF MS, proteomics, Western blot

## Abstract

Brucellosis is a debilitating zoonotic disease that affects humans and animals. The diagnosis of brucellosis is challenging, as accurate species level identification is not possible with any of the currently available serology-based diagnostic methods. The present study aimed at identifying *Brucella* (*B.*) species-specific proteins from the closely related species *B. abortus* and *B. melitensis* using sera collected from naturally infected host species. Unlike earlier reported investigations with either laboratory-grown species or vaccine strains, in the present study, field strains were utilized for analysis. The label-free quantitative proteomic analysis of the naturally isolated strains of these two closely related species revealed 402 differentially expressed proteins, among which 63 and 103 proteins were found exclusively in the whole cell extracts of *B. abortus* and *B. melitensis* field strains, respectively. The sera from four different naturally infected host species, *i.e.*, cattle, buffalo, sheep, and goat were applied to identify the immune-binding protein spots present in the whole protein extracts from the isolated *B. abortus* and *B. melitensis* field strains and resolved on two-dimensional gel electrophoresis. Comprehensive analysis revealed that 25 proteins of *B. abortus* and 20 proteins of *B. melitensis* were distinctly immunoreactive. Dihydrodipicolinate synthase, glyceraldehyde-3-phosphate dehydrogenase and lactate/malate dehydrogenase from *B. abortus*, amino acid ABC transporter substrate-binding protein from *B. melitensis* and fumarylacetoacetate hydrolase from both species were reactive with the sera of all the tested naturally infected host species. The identified proteins could be used for the design of serological assays capable of detecting pan-*Brucella*, *B. abortus*- and *B. melitensis*-specific antibodies.

## 1. Introduction

Brucellosis is a zoonosis affecting a wide range of mammals including humans [1]. The genus *Brucella* currently includes 12 accepted nomo-species, with *Brucella* (*B.*) *abortus* and *B. melitensis* representing the species in the majority of notified human cases. These two species possess strikingly similar genomes [2] but display differences in host specificity and their proteomes [3]. *B. melitensis* is the most virulent species of all brucellae, one of the major causes of abortions in small ruminants and the causative agent of severe infections in humans [4]. *B. abortus* infections occur in cattle while infections in small ruminants and camels are rare [5]. In humans the course of *B. abortus* infections is milder [4].

The conventional methods for species identification include cultivation, as well as genome-based assays [6]. All these methods are hazardous, time-consuming and not suitable for ‘high-throughput analysis’; moreover, the routinely utilized bacterial lipopolysaccharide (LPS)-based serological methods are hampered by cross-reactivity with the LPS of other Gram-negative bacteria such as *Yersinia enterocolitica*, *Salmonella* spp, and *Escherichia coli* O:157 [7]. Furthermore, serological tests cannot distinguish between *B. abortus* and *B. melitensis* infection or between naturally infected and vaccinated animals [8,9].

The aim of this study was to identify *B. abortus*- and *B. melitensis*-specific proteins reacting with circulating antibodies in naturally infected animal host species. This immunoblot-based approach identified several immunodominant proteins from *B. abortus* and *B. melitensis*, which could be used to design a new diagnostic brucellosis assay.

## 2. Results

### 2.1. Comparative Proteomics Analysis of Brucella (B.) abortus and B. melitensis Field Strains

**Figure 1:**
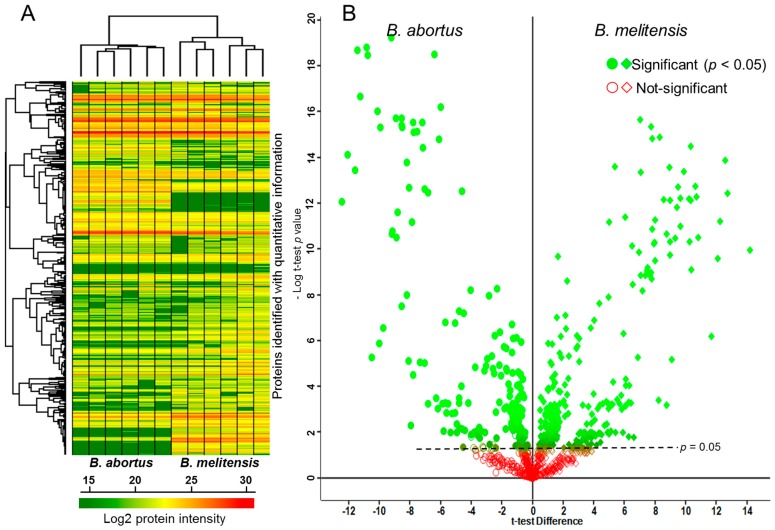
*Brucella* field strain proteome. (**A**) Heat map and hierarchical clustering of 828 quantified proteins from 6 replicates of each field strain; (**B**) Volcano plot comparing the field strains of *B. abortus* and *B. melitensis*. The *p*-values were calculated by unpaired *t*-test analysis using log_2_ transformed peptide intensities. Multiple testing correction was applied [10].

Label-free proteomic analyses involving trypsin digestion, separation of peptides by liquid chromatography (LC) coupled to electrospray ionization, and peptide analysis applying mass spectrometry revealed proteome level differences between *B. abortus* and *B. melitensis* field strains. Figure 1 illustrates the heat map and the hierarchical clustering of the 828 proteins identified in at least three of the six replicates for *B. abortus* and for *B. melitensis*. The volcano plot displays the negative log_10_ *t*-test *p* value over the log_2_ fold change. Proteins with p values above the dotted line (*p* < 0.05) were considered to be differentially expressed between the two *Brucella* species. Initially the two species differed in the expression of 568 proteins (*t*-test significance); upon application of a 1% false discovery rate (FDR) filter [10] the intensities of 402 proteins were still found to be significantly different between *B. abortus* and *B. melitensis*. Of note, 63 of these were found exclusively in the *B. abortus* and 103 exclusively in the *B. melitensis* field strain. The complete list of identified proteins is given in the supplementary table (Supplementary Table S1, sheets 1–4).

### 2.2. Immunoreactive Proteins of B. abortus

**Figure 2:**
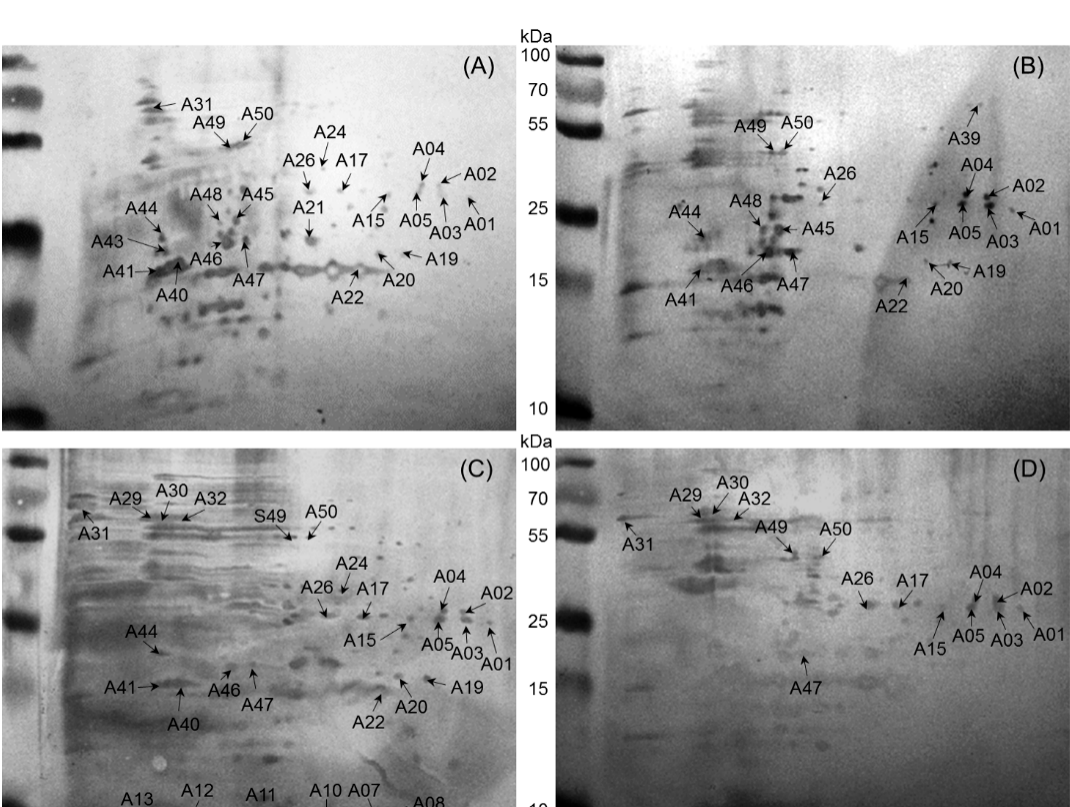
Representative two dimensional electrophoresis immunoblotting images of whole cell proteins from *B. abortus* field strain extracts separated on a 12% polyacrylamide gel. The blot was developed using the TMB (3,3′,5,5′-tetramethylbenzidine liquid substrate) kit after immuno-blotting with serum from (**A**) cattle; (**B**) buffalo; (**C**) sheep; and (**D**) goat and the respective peroxidase-conjugated secondary antibodies.

A total of 50 immunoreactive protein spots, corresponding to different 25 proteins, were detected by two dimensional electrophoresis -immunoblotting with a cell lysate from a *B. abortus* field strain and sera from naturally infected cows, buffaloes, sheep, and goats (Figure 2). Total numbers of proteins identified were 24, 19, 29, and 15 for cow, buffalo, sheep, and goat, respectively. Subsequent Western blot matching revealed 10 spots (A01–05, A15, A26, A47, A49, A50) (bold highlighted in Table 1), which corresponded to five proteins detected in the tested sera from all four naturally infected animal species. There was no unique host-specific immunodominant protein for buffalo and goat, whereas two (A43; A21) and four proteins (A08; A10, A11, A12) were specific for cow and sheep, respectively (Table 1).

### 2.3. Immunoreactive Proteins of B. melitensis

**Figure 3:**
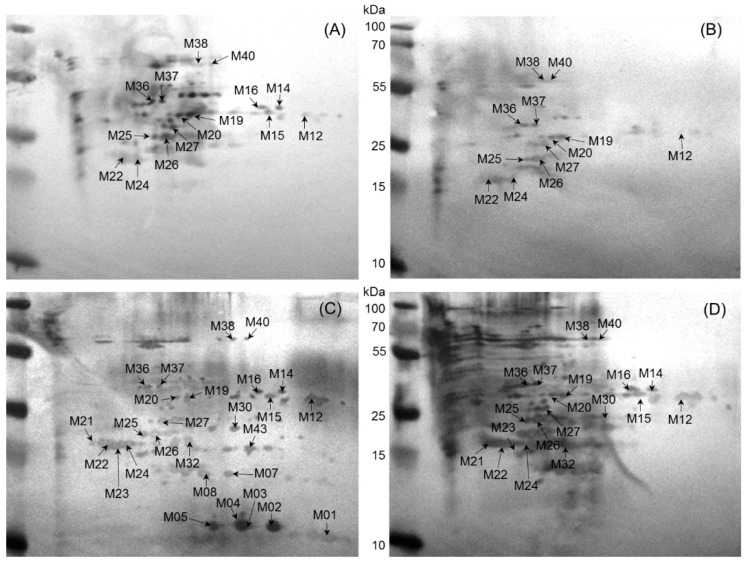
Representative 2D-PAGE immunoblotting images of whole cell proteins from *B. melitensis* field strain extracts separated on a 12% polyacrylamide gel. The blot was developed using the TMB kit after immuno-blotting with serum from (**A**) cattle; (**B**) buffalo; (**C**) sheep; and (**D**) goat and the respective peroxidase-conjugated secondary antibodies.

A total of 43 immunoreactive protein spots corresponding to 20 different proteins were identified in the cell lysate of a *B. melitensis* field strain. Total numbers of proteins identified were 27, 19, 15, and 12 using sera from sheep, goat, cow, and buffalo, respectively (Figure 3). Subsequent Western blot matching revealed 12 spots (M12; M19; M20; M24; M25; M26; M27; M36; M37; M38; M40; M22) (bold highlighted in Table 2) common to all four tested animal species, corresponding to 10 proteins. There was no unique host-specific immunodominant protein for buffalo and cow, whereas three (M32; M21; M23) and six proteins (M01; M02; M05; M07; M08; M43) were specific for sheep and goat, and sheep only (Table 2).

### 2.4. Identification of Cross-Reactive Proteins between B. abortus and B. melitensis

The cell lysates of the *B. abortus* and *B. melitensis* field strains generated a total of 61 immunoreactive spots which could be assigned to 36 proteins. Nine proteins (A47/M25; A22/M43; A41/M36; A45/M27; A40/M24; A07/M01; A10/M05; A13/M12; A21/M21) were detected in cell lysates of *B. abortus* and *B. melitensis* (Table 3), while 16 and 11 proteins were only detected in cell lysates of *B. abortus* or *B. melitensis*, respectively (Table 1 and Table 2). Spot ID A47/M25 (fumarylacetoacetate hydrolase domain-containing protein 2) was found in cell lysates of *B. abortus* and *B. melitensis* and reacted with the sera of all four tested animal species (Table 3 and Table 4). All immunogenic spots reacted only with sera of *Brucella*-positive animals and no reactions were detected with sera from *Brucella*-negative animals.

### 2.5. Comparative Basic Local Alignment Search Tool (BLAST) Analysis

In order to identify similar or identical epitope structures between *Brucella* spp., *Ochrobactrum* spp. and putative cross-reacting bacterial species, five *B. abortus* proteins (spot ID A47; A01; A02; A26; A49) and five *B. melitensis* proteins (spot ID M20; M37; M40; M22; M38) reacting with the sera of all four naturally infected animal host species (*i.e.*, cattle, buffalo, sheep, goat) were selected and submitted to a comparative protein BLAST search (Table 4).

With the exception of the proteins (spot ID) A01, M22, M38, and M40, all proteins displayed identity values ≥95% for *Brucella* spp., *B. suis*, *B. ovis* and *Ochrobactrum* spp. Identity values of all ten proteins with the possibly cross-reacting bacterial species *Y. enterocolitica*, *Y*. *pseudotuberculosis*, *S. enterica* and *E. coli* O:157 were between 26% and 62%.

## 3. Discussion

Diagnosis of brucellosis in veterinary medicine is still a challenging process as it is based on serology and isolation of the agent [6]. The serological assays have their limitations with regard to sensitivity and specificity, moreover, they are not able to distinguish between infected and vaccinated animals [19,20,21]. Hence, the aim of this study was to identify immunodominant proteins in a *B. abortus* and a *B. melitensis* field strain by immunoproteomic screening to detect specific proteins for a future diagnostic assay. Among the 61 proteins found to be immunoreactive, four proteins expressed in *B. abortus*, five proteins expressed in *B. melitensis* cell lysates, and one protein present in both *B. abortus* and *B. melitensis* cell lysates were identified as promising candidates for further analysis.

In contrast to previous studies on the *Brucella* proteome which focussed mainly on vaccine or museum strains with altered immunogenic properties, *i.e.*, diminished or loss of virulence [3,7,12,14,16,19,22,23,24], the present study used a fully virulent *B. abortus* field strain and a fully virulent *B. melitensis* field strain from a naturally infected cow and sheep, respectively. Sera obtained from naturally infected ruminants which had recently aborted and shown strong positive reactions in the RBT, CFT, and ELISA, were subsequently tested against both field strains. The strains and sera used in the present study can therefore be expected to represent the acute phase of the disease. Since naturally infected hosts generally show a stronger immunoreaction than hosts challenged with inactivated antigen [7], it can be assumed that the sera used in the present study contained antibodies against all immunoreactive proteins involved in infection.

In the present study LC/MS-based quantitative proteomic analysis revealed considerable differences in protein expression in the two Brucella field strains. Based on these findings a 2D-PAGE immunoblotting approach was used to determine immunodominant proteins in both field strains. Several studies using two-dimensional gel electrophoresis and mass spectrometry were also able to determine proteome level differences among the laboratory-grown strains *B. abortus* 2308 and *B. melitensis* 16M and revealed a species-specific protein expression pattern [3,14].

The present study identified a total of 61 immunoreactive protein spots from the proteomic profiles of *B. abortus* and *B. melitensis* using MALDI-TOF MS and the NCBI database search corresponding to 36 proteins. When performing a data base search against the sequence information of all entries in the NCBI database, the likelihood of identifying suitable proteins was significantly increased by applying MS/MS matched to at least one unique peptide. This approach contrasts that of Al-Dahouk *et al.*, Yang *et al.* and Connolly *et al.*, [7,12,16], who searched only against data sets of the *Brucella* species used in their experiments, and Zhao *et al*. [22], who selected proteins containing more than five peptide matches.

Each *Brucella* species can be associated with a specific host, *i.e.*, *B. abortus* usually infects bovines, whereas *B. melitensis* is the most predominant species in sheep and goat [5]. Despite the close genetic relationship among *Brucella* spp. one could speculate that certain proteins induce a host species-specific immunoreaction. This hypothesis is corroborated by the findings of Zhao *et al.* [22], who demonstrated that some proteins are themselves immunogenic and induce high immunogenicity in the host species but not in others. The sera obtained from sheep were the most reactive, with 56 identified immunogenic protein spots, whereas 39, 31, and 34 spots were found in the sera of cow, buffalo and goat, respectively. Previous studies using the same immunoproteomic techniques as in the present study identified a differing range of immunoreactive proteins in various *Brucella* spp. and animal species [7,12,16,19,22,23]. These observed differences can be attributed to the technical procedures during protein preparation and the source/type of sera samples used, *i.e.*, field or experimental, early or late stage of infection [23]. Moreover, these findings are indicative of a host species-specific immunoreaction.

Ten immunogenic proteins specific either from *B. abortus* (*n* = 4), *B. melitensis* (*n* = 5) or both (*n* = 1) were reactive with the sera of all four tested host species, *i.e.*, cattle, buffalo, sheep, and goat. The mitochondrial catalytic enzyme, fumarylacetoacetate hydrolase domain-containing protein (FAHD2) was found in both *B. abortus* and *B. melitensis* cell lysates. To date, the role of this mitochondrial protein in the pathogenesis of *Brucella* infections is not known. Four proteins were identified in *B. abortus* only, *i.e.*, dihydrodipicolinate synthase (DHDPS), glyceraldehyde-3-phosphate dehydrogenase (GAPDH), lactate/malate dehydrogenase, and phosphopyruvate hydratase. DHDPS is essential for bacterial growth and involved in the lysine biosynthesis pathway. This protein has been isolated from various Gram positive and Gram negative bacteria and is considered to be an attractive antibiotic target [25]. GAPDH is a protein of the *Brucella*-containing vacuole (BCV) and essential for *B. abortus* virulence [26]. Studies using recombinant *B. abortus* derived GAPDH induced both humoral and cellular immune responses during experimental infection with *B. abortus* in natural hosts (cattle and sheep) and mice [11]. However, when used as DNA vaccine it provided only partial protection against experimental *B. abortus* infection in mice [11]. lactate/malate dehydrogenase from *B. abortus* is considered to be a promising candidate for serodiagnosis and vaccine development due to its immunogenic characteristics [27], but further studies are required. Phosphopyruvate hydratase proteins participate in glycolysis, but their importance as possible diagnostic candidates is not known [28].

Five proteins found only in *B. melitensis* cell lysates were immunoreactive in all four host species: thiosulfate-binding protein precursor, which specifically binds thiosulfate and is involved in its transmembrane transport; amidohydrolase 3, a member of the amidohydrolase superfamily. These proteins catalyse the hydrolysis of amide or amine bonds in a large number of different substrates [29]. Amino-acid ABC transporter substrate-binding protein, a transmembrane protein previously found via proteome analysis in *B. melitensis* and *B. ovis* [30], and two hypothetical proteins closely related to the ABC transporter substrate-binding proteins. The function of these differentially expressed proteins in natural *B. melitensis* infection is not known to date.

LPS is the major cell surface antigen of *Brucella* and one of the main reasons for serological cross-reactions with other Gram-negative bacteria such as *E. coli*, *Salmonella* spp*.*, and *Y. enterocolitica* [7,21,31]. LPS has been shown to bind to sodium dodecyl sulfate (SDS), and may mimic protein spots in SDS polyacrylamide gels [32,33]. LPS closely associates with the protein and traces of LPS are expected to be present in the whole cell protein extract irrespective of the extraction method used. In order to exclude the likelihood of LPS interference during immunostaining, the LPS concentration was measured in the extract. The whole cell protein extracts of *B. abortus* and *B. melitensis* contained 0.9 and 100 ng/mL of LPS, respectively. This LPS concentration is below the limit of detection of monoclonal antibodies [34] and therefore any interference of LPS can be excluded.

BLAST search to assess the similarity of these immunoreactive proteins identified among several *Brucella* species and possibly cross-reacting bacteria revealed that by combining various proteins it is possible to design a pan-*Brucella* test as well as a species-differentiating assay. Glyceraldehyde-3-phosphate dehydrogenase, lactate/malate dehydrogenase, thiosulfate-binding protein precursor, the amino acid ABC transporter substrate-binding proteins, and FAHD2 are suitable candidates for designing a pan-*Brucella* test. Aminohydrolase 3 on the other hand, might be useful for the differentiation of *B. ovis* and *Ochrobactrum* spp. from *B. abortus*, *B. melitensis*, and *B. suis*.

## 4. Materials and Methods

### 4.1. Bacterial Strains and Sera Selection

The strains and sera used in the present study were obtained from the Friedrich-Loeffler-Institut (FLI), Federal Research Institute for Animal Health, Institute of Bacterial Infections and Zoonoses, Jena, Germany. The utilised *Brucella* field strains were isolated from an outbreak in Turkey (*B. abortus*; cow) and an outbreak in China (*B. melitensis*; ewe). Identification and biotyping of these *Brucella* isolates was carried out as previously described [6,35]. The serum samples were collected in Egypt from naturally infected ruminants which had recently aborted (*i.e.*, acute stage of infection); 35 sera were sent to the FLI for further analysis. Sera were considered positive if the Rose Bengal test (RBT) showed strong agglutination; the complement fixation test (CFT) showed more than 1000 sensE/mL; and the ELISA an OD ≥ 3. For each animal species (*i.e.*, cattle, buffalo, sheep, goat) three positive serum samples were selected and used separately in the present study [6]. Negative serum samples were collected from non-infected animals in a non-endemic region. Experiments were run in three replicates. No sera could be obtained from the outbreaks in Turkey and China, and no isolates could be obtained from the outbreak in Egypt.

### 4.2. Ethics Statement

The sera samples collected during routine diagnosis from Egypt were approved (no 11/2012) by the Ethics committee at the Dean’s office, Faculty of Veterinary Medicine, Benha University, Ministry of Higher Education, Qalyobia, Egypt. All further sera used in this study were samples originally submitted for diagnostic purposes to and subsequently stored at FLI. According to German law, ethical approval or special permission concerning animal welfare is not required for sera meant for diagnostic purposes.

### 4.3. Cell Culture and Protein Extraction

Whole cell protein of the *B. abortus* and *B. melitensis* field strains was extracted as described previously [36]. The strains were cultured in Tryptic Soy media for 48 h at 37 °C; bacteria were harvested by centrifugation, washed twice with phosphate buffer saline, spun down, and the resulting pellets resuspended in 80% ethanol (*v*/*v*). Following centrifugation, the ethanol was discarded and the cell pellet air dried to ensure the removal of traces of ethanol. The cell pellets were then resuspended in 250 µL of HEPES buffer (20 mM, pH 7.4) and sonicated (duty cycle: 1.0, amplitude: 100%, UP100H; Hielscher Ultrasound Technology, Teltow, Germany) for 45 s on ice. After centrifugation at 11,290× *g* for 10 min at 4 °C, the clear supernatant was collected. The protein content was determined using the modified Bradford method (Bio-Rad, München, Germany) [37]. The concentration of LPS was determined as concentration of endotoxin using the recombinant factor C fluorescence assay (Haemotox rFC Haemochrom Diagnostica GmbH, Essen, Germany). The sample was spiked with an endotoxin standard (PPC-Spike) and LPS determination was carried out according to the manufacturer’s instructions.

### 4.4. In-Solution Trypsin Digestion

Ten µg of the protein extract (*B. abortus* and *B. melitensis*) were acetone precipitated and reconstituted in 20 µL of denaturation buffer (6 M urea/2 M thiourea in 10 mM HEPES, pH 8.0). The following steps were carried out with incubation steps at room temperature and gentle shaking. Reduction of disulfide bridges was performed by adding 0.2 µL of 10 mM dithiothreitol in 50 mM of ammonium bicarbonate (ABC) and incubation for 30 min, followed by an alkylation step with 0.4 µL of 55 mM iodacemtamide in ABC and incubation in the dark for 20 min. Then 0.4 µL of LysC (Sigma, Taufkirchen Germany) solution (0.5 µg/µL in ABC) was added and subjected to overnight incubation at RT. After pre-digestion with LysC, the sample was diluted by adding 75 µL of ABC to bring down the urea concentration to <2 M and digestion was started by adding 0.4 µL of 0.5 µg/µL trypsin in 50 mM ABC. Following overnight incubation, trypsin activity was stopped by adding 100 µL of 5% acetonitrile in 3% trifluoroacetic acid.

### 4.5. Liquid Chromatography-Electrospray Ionization-Tandem Mass Spectrometry (LC-ESI-MS/MS)

After digestion peptide samples were desalted by solid phase extraction (SPE), using C_18_ stage tips [38]. Desalted peptide mixtures were separated by reverse-phase chromatography using a Dionex Ultimate 3000 nanoLC on in-house manufactured 25 cm fritless silica microcolumns with an inner diameter of 100 µm. Columns were packed with ReproSil-Pur C_18_-AQ 3 µm resin (Dr. Maisch GmbH, Ammerbuch-Entringen, Germany). Peptides were separated on a 5%–60% acetonitrile gradient with 0.1% formic acid at a flow rate of 350 nL/min for 90 min. Eluting peptides were ionized on-line by electrospray ionization and transferred into an LTQ Orbitrap Velos mass spectrometer (Thermo Fisher Scientific, Bremen, Germany). The LTQ-Orbitrap was operated in the positive mode to simultaneously measure full scan MS spectra (from *m*/*z* 300–1700) in the Orbitrap analyzer at resolution *R* = 60,000 following isolation and fragmentation of the twenty most intense ions in the LTQ part by collision-induced dissociation.

### 4.6. Protein Identification and Data Analysis

A freely available software suit, MaxQuant (version. 1.3.0.5) (Max-Planck-Institute of Biochemistry, Martinsried, Germany) was used to process the raw MS files and the search engine, Andromeda [39] was utilized to search the peak list files against forward and backward protein sequences of *Brucella* (Reference proteome *Brucella abortus* (strain 2308) with protein count of 3022 and *Brucella melitensis* (strain M28) with a protein count of 3351) downloaded from Uniprot database and 248 frequently observed laboratory contaminants (accession date: August 2015). Initial maximum precursor and fragment mass deviations were set to 7 ppm and 0.5 Da, respectively. Methionine oxidation/acetylation of peptide N-termini and cysteine carbamidomethylation were set as variable and fixed modification, respectively, for the search. Furthermore, enzyme specificity was set to trypsin and a maximum of two missed cleavages was allowed for searching. The target-decoy-based false discovery rate (FDR) for peptide and protein identification was set to 1% for peptides and proteins and the minimum peptide length was set to 7 amino acids. Precursor mass tolerance was set to 20 ppm. The mass tolerance for fragment ions was set to 0.5 Da. MS-quantification of proteins was performed using the label-free quantification algorithm of the MaxQuant software package (Max-Planck-Institute of Biochemistry, Martinsried, Germany) [40].

The statistical analysis was performed by unpaired Student´s *t* Test using the Perseus software [41] version 1.4.1.3 package (Max-Planck-Institute of Biochemistry, Martinsried, Germany). For FDR ccorrections of the significant *p*-values (*p* <0.05) the Benjamini-Hochberg procedure [10] was applied. Heat map and volcano plots were calculated for further assessment and visualization.

### 4.7. Two-Dimensional Electrophoresis

Two-dimensional electrophoresis (2D-PAGE) was performed as described [42]. Briefly, the first dimension of 2D-PAGE was performed by applying 100 µg of acetone-precipitated protein per sample to pI, 4–7, 7.0 cm immobilized pH gradient (IPG) strips (Immobiline™ Dry Strip, GE Healthcare Bio-sciences AB; Uppsala, Sweden). The strips were rehydrated overnight at room temperature with 135 µL DeStreak Rehydration Solution. Isoelectric focusing (IEF) was performed by using the Ettan™ IPGphor3™ Unit (GE Healthcare Europe; Freiburg, Germany) and carried out at 20 °C for 6.5 h at 5000 V and 50 µA/strip.

Then the strips were sequentially equilibrated for 20 min in 2 mL equilibration buffer 1 (0.05 M trichloroethylene HCl (pH 8.8), 6 M urea, 30% glycerol, 4% SDS, 2% DTE, 0.002% bromophenol blue) and equilibration buffer 2 (0.05 M trichloroethylene HCl (pH 8.8), 6 M urea, 30% glycerol, 4% SDS, 2.5% iodoacetamid, 0.002% bromophenol blue). Standard molecular weight prestained protein ladder marker (10–250 kDa; Page Ruler™ Plus, ThermoScientific; Germany) and IPG strips were loaded onto homogeneous 12% polyacrylamide gels and sealed with 1% agarose solution. Electrophoresis was carried out at room temperature and 10 mA/gel until the tracking dye reached the bottom of the gels (1.5 h). 2D-PAGE protein profiles were visualized using Coomassie blue stain as previously described [43].

### 4.8. 2-D-PAGE Western Blotting

2D-PAGE Immunoblotting was carried out as previously described [19,36] with minor modifications. Briefly, proteins were separated on 2D-PAGE gels and transferred at 80 mA/gel for 90 min to nitrocellulose membranes (0.2 µM Bio-Rad laboratories; München, Germany) using Towbin transfer buffer (0.025 M Tris, 0.192 M glycine, 2.33% SDS, 20% (*v*/*v*) methanol, pH 8.3). The nitrocellulose membrane was blocked overnight at room temperature with gentle shaking in 1% skimmed milk in Tris buffered saline (TBS). The membrane was washed twice using TBS with Tween (TBST; 20 mM Tris, pH 7.5; 500 mM NaCl; 0.05% Tween-20; 10 min). Next, the nitrocellulose membrane was placed for 90 min at room temperature in a diluted solution of the respective sera in TBST. Bovine sera (1:200 dilution) and small ruminants sera (1:5000 dilution) were used as primary antibody source while 1:1000 diluted anti-bovine IgG (H and L) (Chicken) peroxidase-conjugated, anti-sheep IgG (H and L) (Donkey) peroxidase-conjugated and anti-goat IgG (H and L) (Chicken) peroxidase-conjugated antibody served as secondary antibody source. All the secondary antibodies were obtained from Biomol-Rockland, Hamburg, Germany. After washing the nitrocellulose membrane twice with TBST for 10 min, the detection of signals was carried out using the TMB kit™ (3,3′,5,5′-tetramethylbenzidine liquid substrate; Sigma-Aldrich; Steinheim, Germany) according to the manufacturer’s description.

### 4.9. In-Gel Trypsin Digestion and Matrix-Assisted Laser Desorption/ionization-Tine of Flight-Mass Spectrometry (MALDI-TOF MS/MS)

Following the selection of the spots of interest, *i.e.*, spots detected in all three replicates, the protein spots corresponding to the Western blots were excised from the gel, destained, and subjected to overnight trypsin digestion (0.01 µg/µL) (Promega; Mannheim, Germany) as previously described [44]. The digested precipitates were reconstituted in 3.5 µL 5% acetonitrile in 0.1% TFA (trifluoroacetic acid; Merck; Darmstadt, Germany). The reconstituted precipitates were then spotted on to target plates for matrix-assisted laser desorption/ionization-time-of-flight mass spectrometry (MALDI-TOF MS) on a Bruker Ultraflex II instrument (Bruker Daltonik; Bremen, Germany) using HCCA (α-cyano-4-hydroxycinnamic acid; Sigma-Aldrich; Steinheim, Germany) as matrix. A database search was conducted against all entries using the MS/MS ion search mode (MASCOT, http://www.matrixscience.com) as previously described [36]. Protein identification was considered valid if more than two peptides matched and the MOWSE score was significant (*p* < 0.05).

### 4.10. Comparison of the Identified Proteins and Other Cross-Reactive Bacteria

BLAST search was done as previously described [3] to compare the identified proteins against *Brucella* spp., *B. suis*, *B. ovis*, *Ochrobactrum* spp., *Y. enterocolitica*, *Y. pseudotuberculosis*, *S. enterica*, and *E. coli* O:157, the latter five species being the most cross-reactive bacteria with *Brucella*. Query cover and identity values were evaluated and cut-off values set between 31%–54%.

## 5. Conclusions

The sera from naturally infected host species appear to possess different antibodies against *B. abortus* and *B. melitensis*. The presence of specific antibodies against four proteins of *B. abortus*, fumarylacetoacetate hydrolase 2, dihydrodipicolinate synthase, glyceraldehyde-3-phosphate dehydrogenase, and lactate/malate dehydrogenase as well as one protein from *B. melitensis*, ABC transporter substrate-binding protein, indicates that these proteins might be useful for designing a serology-based assay for the rapid species determination of *Brucella*. As suggested earlier, these results should be further verified using bacterial strains and sera collected from various geographical regions and sera from a variety of host species.

Cross-reactivity with other Gram negative bacteria and within the species of the genus is the major hindrance for the serological diagnosis of brucellosis. The results presented here open up new possibilities for the serodiagnosis of brucellosis by providing *Brucella* species-specific immunodominant protein candidates reacting only with sera collected from naturally infected cattle, buffaloes, sheep, and goats. The study provides information on new protein candidates and could help to improve the serological diagnosis of brucellosis.

## Figures and Tables

**Table 1:** Immunoreactive proteins from *Brucella* (*B.*) *abortus* using two dimensional electrophoresis Western blot and Matrix-assisted laser desorption/ionization-Tine of Flight-Mass Spectrometry (MALDI-TOF-MS). Spot ID: Spot identification; A: *B. abortus*; M: *B. melitensis*; Acc.ID: Accession number at the National Center for Biotechnology Information (NCBI) databank; Proteins: Description as in the NCBI database and the bold highlighted proteins were those considered as a promising candidate for future sero-diagnostics assays; *M*_w_: Molecular weight; MOlecular Weight Search (MOWSE) score: −10*Log (*p*), where *p* is the probability that the observed match is a random event. This list includes only bands with a MOWSE score greater than (*p* <0.05); pI: Isoelectric point; Sequence coverage (%): Is the percentage of peptide that covers the complete sequence of the protein; No. of peptides matching: Is the number of peptides that matched with the protein sequence.

No.	Spot ID	Acc.ID	Protein	*M*_w_	MOWSE Score ^a^	pI	Sequence Coverage (%)	No. of Peptides Matching	Host	Reference/Cross-Matching Spot ID
1	**A01**	gi|256369084	**Dihydrodipicolinate synthase**	31,892	244	6.26	67	13	Cow, Buffalo, Sheep, Goat	
	**A03**	gi|256369084	**Dihydrodipicolinate synthase**	31,892	426	6.26	67	13	Cow, Buffalo, Sheep, Goat	
	**A05**	gi|493692811	**Dihydrodipicolinate synthase**	33,539	132	7.08	68	14	Cow, Buffalo, Sheep, Goat	
	**A15**	gi|495149454	**Dihydrodipicolinate synthase**	31,753	75	5.94	28	5	Cow, Buffalo, Sheep, Goat	
2	**A02**	gi|496823699	**Glyceraldehyde-3-phosphate dehydrogenase**	36,385	356	6.26	48	13	Cow, Buffalo, Sheep, Goat	[10]
	**A04**	gi|4165122	**Glyceraldehyde-3-phosphate dehydrogenase**	36,344	107	5.89	38	8	Cow, Buffalo, Sheep, Goat	[10]
3	**A26**	gi|226887955	**Lactate malate dehydrogenase**	34,152	243	5.24	31	5	Cow, Buffalo, Sheep, Goat	
4	**A47**	gi|493015116	**Hypothetical protein (fumarylacetoacetate hydrolase family protein)**	29,383	343	5.09	36	7	Cow, Buffalo, Sheep, Goat	M25
5	**A49**	gi|17987134	**Phosphopyruvate hydratase**	45,462	421	4.99	53	18	Cow, Buffalo, Sheep, Goat	
	**A50**	gi|148560469	**Phosphopyruvate hydratase**	45,431	494	5.03	47	16	Cow, Buffalo, Sheep, Goat	
6	A20	gi|148558534	Metal-dependent hydrolase	25,257	103	5.58	44	7	Cow, Buffalo, Sheep	
7	A17	gi|82700282	Choloylglycine hydrolase	36,868	108	5.62	29	9	Cow, Sheep, Goat	
8	A22	gi|490830157	Hydrolase	27,731	134	6.07	50	8	Cow, Buffalo, Sheep	[11] M43
	A19	gi|490830157	Hydrolase	27,731	383	6.07	48	8	Cow, Buffalo, Sheep	[11]
9	A44	gi|320161003	Putative DNA processing protein	40,919	56	5.85	42	7	Cow, Buffalo, Sheep	
10	A46	gi|489055332	2-Hydroxyhepta-2,4-diene-1,7-dioate isomerase	30,092	380	5.08	48	8	Cow, Buffalo, Sheep	
11	A41	gi|493691811	Sugar ABC transporter substrate-binding protein	33,258	440	5.11	52	11	Cow, Buffalo, Sheep	M36
12	A45	gi|384211119	Lysine-arginine-ornithine-binding periplasmic protein	36,684	440	5.09	57	15	Cow, Buffalo	M27
13	A48	gi|152013695	ADP/ATP translocase	20,876	63	9.63	31	4	Cow, Buffalo	
14	A39	gi|62317242	Urocanate hydratase	61,589	173	6.04	19	11	Buffalo, Sheep	
15	A24	gi|493147262	Sulfate transporter subunit	37,727	132	5.92	38	11	Cow, Sheep	
16	A31	gi|493053174	Catalase	55,556	223	6.62	36	18	Cow, Goat	
17	A40	gi|17988780	d-ribose-binding periplasmic protein precursor	31,030	193	5.60	57	9	Cow, Sheep	[12,13,14] M24
18	A29	gi|148558491	Chaperonin GroEL	57,505	99	5.08	18	7	Sheep, Goat	[7,15,16,17]
	A30	gi|14855849	Chaperonin GroEL	57,505	92	5.08	20	9	Sheep, Goat	[7,15,16,17]
19	A32	gi|144108	Heat shock protein	57,534	94	5.33	27	12	Sheep, Goat	
20	A43	gi|492987884	Protein grpE	24,883	128	4.70	40	12	Cow	
21	A07	gi|384446825	Superoxide dismutase, copper/zinc binding protein	17,255	370	6.10	64	7	Sheep	[7,16,17,18] M01
	A8	gi|489058379	Superoxide dismutase copper/zinc binding protein	18,205	242	6.24	54	5	Sheep	[7,16,17,18]
22	A10	gi|17989230	19 kDa periplasmic protein	20,238	68	6.06	8	1	Sheep	M05
23	A11	gi|222447132	Ferritin (bacterioferritin)	20,895	68	6.05	33	5	Sheep	
	A13	gi|222447132	Ferritin (bacterioferritin)	20,895	183	5.05	36	4	Sheep	M12
24	A12	gi|493690773	Bacterioferritin, partial	16,118	220	4.81	33	3	Sheep	[16,17]
25	A21	gi|89258175	31 kDa cell surface protein	31,084	293	5.50	38	9	Cow	[16] M21

**Table 2:** Immunoreactive proteins from *B. melitensis* using 2D-PAGE Western blot and MALDI-TOF-MS. Spot ID: Spot identification; A: *B. abortus*; M: *B. melitensis*; Acc.ID: Accession number at NCBI; sequence in NCBI databank; Proteins: Description as in the NCBI database and the bold highlighted proteins were those considered as a promising candidate for future sero-diagnostics assays; *M*_w_: Molecular weight; MOWSE score: −10*Log (*p*), where *p* is the probability that the observed match is a random event. This list includes only bands with a MOWSE score greater than (*p* <0.05); pI: Isoelectric point; Sequence coverage (%): Is the percentage of peptide that covers the complete sequence of the protein; No. of peptides matching: Is the number of peptides that matched with the protein sequence.

No.	Spot ID	Acc.ID	Protein	*M*_w_	MOWSE Score ^a^	pI	Sequence Coverage (%)	No. of Peptides Matching	Host	Reference/Cross-Matching Spot ID
1	**M12**	gi|222447132	**Ferritin (Bacterioferritin)**	20,895	183	5.05	36	4	Sheep, Goat, Cow, Buffalo	A13
2	**M19**	gi|225852817	**Sulfate ABC transporter substrate-binding protein**	37,151	324	5.51	44	12	Sheep, Goat, Cow, Buffalo	
3	**M20**	gi|17986956	**Thiosulfate-binding protein precursor**	37,152	34	5.31	5	1	Sheep, Goat, Cow, Buffalo	
4	**M24**	gi|17988780	**d-ribose-binding periplasmic protein precursor**	31,030	280	5.60	29	5	Sheep, Goat, Cow, Buffalo	[12,13,14] A40
5	**M25**	gi|225851771	**Fumarylacetoacetate hydrolase domain-containing protein 2**	30,118	471	5.00	61	11	Sheep, Goat, Cow, Buffalo	A47
	**M26**	gi|225851771	**Fumarylacetoacetate hydrolase domain-containing protein 2**	30,118	492	5.00	56	11	Sheep, Goat, Cow, Buffalo	
6	**M27**	gi|384211119	**Lysine-arginine-ornithine-binding periplasmic protein**	36,684	240	5.09	31	10	Sheep, Goat, Cow, Buffalo	A45
7	**M36**	gi|516360216	**Sugar ABC transporter substrate-binding protein, partial**	44,963	121	5.15	50	15	Sheep, Goat, Cow, Buffalo	A41
8	**M37**	gi|493172683	**Amino acid ABC transporter substrate-binding protein**	31,331	178	5.24	48	7	Sheep, Goat, Cow, Buffalo	[16]
9	**M40**	gi|384410242	**Amidohydrolase 3**	63,567	265	5.47	42	20	Sheep, Goat, Cow, Buffalo	
10	**M22**	gi|493003797	**Hypothetical protein similar to amino acid ABC transporter substrate-binding protein**	21,946	90	5.06	33	4	Sheep, Goat, Cow, Buffalo	
	**M38**	gi|493155701	**Hypothetical protein similar to ABC transporter substrate-binding protein**	58,947	437	4.97	43	24	Sheep, Goat, Cow, Buffalo	
	M32	gi|492818336	Hypothetical protein similar to ABC transporter substrate-binding protein	31,905	113	5.57	33	7	Sheep, Goat	
11	M14	gi|490823297	Alcohol dehydrogenase	36,537	116	6.07	21	7	Sheep, Goat, Cow	
	M16	gi|489059662	Alcohol dehydrogenase	43,149	99	7.66	25	9	Sheep, Goat, Cow	
12	M15	gi|493009422	Thiamine-binding periplasmic protein	36,829	164	5.71	43	9	Sheep, Goat, Cow	
13	M21	gi|89258175	31 kDa cell surface protein	31,084	96	5.50	52	10	Sheep, Goat	[16] A21
	M30	gi|89258175	31 kDa cell surface protein	31,084	166	5.50	16	5	Sheep, Goat	[16]
14	M23	gi|225686619	rhizopine-binding protein	33,294	257	5.11	55	11	Sheep, Goat	
15	M01	gi|384446825	Superoxide dismutase, copper/zinc binding protein	17,255	222	6.10	51	6	Sheep	[7,16,17,18] A07
	M03	gi|384446825	Superoxide dismutase, copper/zinc binding protein	17,255	86	6.1	57	6	Sheep	[7,16,17,18]
16	M02	gi|118137288	Copper/zinc superoxide dismutase	16,176	297	6.11	63	7	Sheep	
	M04	gi|551701922	Copper/zinc superoxide dismutase	16,176	83	6.11	61	6	Sheep	
17	M05	gi|384446516	19 kDa periplasmic protein	18,735	219	5.65	20	4	Sheep	A10
18	M07	gi|495782928	Transaldolase	23,554	264	5.47	41	8	Sheep	[18]
19	M08	gi|493009465	Fructose-6-phosphate aldolase	23,554	244	5.47	22	5	Sheep	
20	M43	gi|490830157	Hydrolase	27,731	371	6.07	48	8	Sheep	A22

**Table 3:** Cross-reactive proteins identified by immunoblotting in the *B. abortus* and *B. melitensis* field strains using sera from different naturally infected host species (A: *B. abortus*; M: *B. melitensis*); Acc.ID: accession number at NCBI.

No.	Acc.ID	Protein	*B. abortus*		*B. melitensis*	
			Spot ID	Host	Spot ID	Host
1	gi|493015116	Fumarylacetoacetate hydrolase family protein	A47	Cow, Buffalo, Sheep, Goat	M25	Cow, Buffalo, Sheep, Goat
2	gi|490830157	Hydrolase	A22	Cow, Buffalo, Sheep	M43	Sheep
3	gi|493691811	Sugar ABC transporter substrate-binding protein	A41	Cow, Buffalo, Sheep	M36	Cow, Buffalo, Sheep, Goat
4	gi|384211119	Lysine-arginine-ornithine-binding periplasmic protein	A45	Cow, Buffalo	M27	Cow, Buffalo, Sheep, Goat
5	gi|17988780	d-ribose-binding periplasmic protein precursor	A40	Cow, Sheep	M24	Cow, Buffalo, Sheep, Goat
6	gi|384446825	Superoxide dismutase, copper/zinc binding protein	A07	Sheep	M01	Sheep
7	gi|384446516	19 kDa periplasmic protein	A10	Sheep	M05	Sheep
8	gi|222447132	Ferritin (Bacterioferritin)	A13	Sheep	M12	Cow, Buffalo, Sheep, Goat
9	gi|89258175	31 kDa cell surface protein	A21	Cow	M21	Sheep, Goat

**Table 4:** Comparative Blast search between the identified proteins obtained from *B. abortus* and *B. melitensis* field strains and from proteins of putatively cross-reacting bacteria (A: *B. abortus*; M: *B. melitensis*); low cross-reactivity % values are in bold text. Acc.ID: Accession number at NCBI; Y: *Yersinia*; S: *Salmonella.*

No.	Spot ID	Acc.ID	Protein	Locus, Query Cover (QC) and Identity (I)									Host
				100% Identity	*Brucella* spp.	*B. suis*	*B. ovis*	*Ochrobactrum* spp.	*Y. enterocolitica*	*Y. pseudotuberculosis*	*S. enterica*	*E. coli* O:157	
1	A47/M25	gi|493015116 MW 29383	FAHD2	WP_006093223 *B. Abortus* QC 100% I 100%	WP_006162877 QC 80% I 96%	WP_006200925 QC 100% I 96%	YP_001258270 FAHD QC 100% I 96%	WP_006470802 QC 100% I 92%	Not found 16.04.2014	YP_001401380 FAHD QC 98% I 62%	YP_001588666 QC 78% I 41%	Not found 16.04.2014	Cow, Buffalo, Sheep, Goat
2	A01	gi|256369084 MW 31892	Dihydrodi-picolinate synthase	YP_003106592 *B. microti* CCM 4915	WP_006165259 QC 100% I 99%	NP_697660 QC 100% I 99%	YP_001257393 QC 98% I 29%	WP_021587874 QC 100% I 95%	YP_006003506 QC 99% I 46%	YP_071290 QC 99% I 46%	WP_023259918 QC 99% I 45%	NP_311367 QC 99% I 45%	Cow, Buffalo, Sheep, Goat
3	A02	gi|496823699 MW 36385	Glyceraldehyde-3-phosphate dehydrogenase	WP_009374365 *Brucella* spp. QC 100% I 100%	WP_009374365 QC 100% I 100%	NP_698712 QC 100% I 99%	NP_698712.1 QC 100% I 99%	WP_021588015 QC 100% I 96%	WP_019083593Q C 98% I 54%	YP_071698 QC 99% I 46%	WP_000218344 QC 99% I 46%	ELW37260 QC 97% I 52%	Cow, Buffalo, Sheep, Goat
4	A26	gi|226887955 MW 34152	Lactate malate dehydrogenase	WP_002970355 *B. abortus* QC 98% I 100%	3GVH_A *B. melitensis* QC 100% I 100%	YP_001628354 QC 98% I 99%	YP_001259751 QC 98% I 99%	WP_007872232 L/M dehydrogenase QC 98% I 98%	WP_019080697 QC 67% I 33%	YP_069003 QC 67% I 33%	YP_218284.1 QC 86% I 31%	ELV66131 QC 86% I 31%	Cow, Buffalo, Sheep, Goat
5	A49	gi|17987134 MW 45462	Phosphopyruvate hydratase	NP_539768 *B. melitensis* QC 100% I 100%	YP_008839865 QC 100% I 99% enolase	NP_698137 QC 100% I 99%	YP_001259054 QC 100% I 99%	YP_001370601 QC 100% I 97%	YP_001005091. QC 99% I 60%	YP_069296.1 QC 99% I 60%	WP_016735109 QC 99% I 61%	ELV67289 QC 99% I 61%	Cow, Buffalo, Sheep, Goat
6	M20	gi|17986956 MW 37152	Thiosulfate-binding protein precursor	NP_539590.1 *B. melitensis* QC 100% I 100%	WP_008934207 QC 100% I 99%	WP_020628554 QC 100% I 100%	YP_001259236 QC 100% I 99%	WP_021586689 QC 100% I 91%	AHM75213.1 QC 98% I 55%	YP_071244.1 QC 99% I 55%	WP_000290287 QC 93% I 57%	NP_288986 QC 92% I 57%	Sheep, Goat, Cow, Buffalo
7	M37	gi|493172683 MW 31331	Amino acid ABC transporter substrate-binding protein	WP_004685846 *B. melitensis* QC 100% I 94%	WP_006161567 *Brucella* spp QC 100% I 95%	NP_698767 putative branch QC 100% I 95%	NP_698767 putative branch QC 100% I 95%	WP_006467797 QC 100% I 90%	WP_019080170 QC 95% I 40%	Not found 16.04.2014	WP_000822979 leucine branch QC 95% I 40%	ELV65532 leucine specific QC 95% I 42%	Sheep, Goat, Cow, Buffalo
8	M40	gi|384410242 MW 63567	Amidohydrolase 3	YP_005602224 *B. melitensis* M5 QC 100% I 100%	YP_005114197 *B. abortus* QC 100% I 99%	WP_004689025 QC 100% I 99%	YP_001257534 amidohydrolase QC 88% I 31%	YP_001371888 QC 100% I 53%	Not found 16.04.2014	Not found 16.04.2014	WP_023220860 amidohydrolase QC 90% I 26%	Not found 16.04.2014	Sheep, Goat, Cow, Buffalo
9	M22	gi|493003797 MW 21946	Hypothetical protein (amino acid ABC transporter substrate-binding protein)	WP_023080384 *B. melitensis* QC 100% I 100%	WP_006085596 *B. abortus* QC 100% I 100%	WP_023080435 QC 100% I 84%	YP_001258837 ABC transporter QC 100% I 100%	WP_006466755 ABC transporter QC 99% I 94%	YP_001006291. ABC transporter QC 98% I 39%	Not found 16.04.2014	Not found 16.04.2014	Not found 16.04.2014	sheep, goat, cow, buffalo
10	M38	gi|493155701 MW 58947	Hypothetical protein (ABC transporter substrate-binding protein)	WP_006256535 *B. melitensis* QC 100% I 99%	WP_006164780 QC 100% I 100%	WP_006197818 QC 100% I 99%	WP_006157758 QC 99% I 70%	WP_010658797 ABC transporter QC 100% I 89%	WP_019083182 ABC transporter QC 97% I 40%	Not found 16.04.2014	WP_023210061 ABC transporter QC 93% I 40%	Not found 16.04.2014	sheep, goat, cow, buffalo

## Supplementary Materials

Supplementary materials can be found at http://www.mdpi.com/1422-0067/17/5/659/s1.
